# The dual role of platelet‐innate immune cell interactions in thrombo‐inflammation

**DOI:** 10.1002/rth2.12266

**Published:** 2019-10-17

**Authors:** Julie Rayes, Joshua H. Bourne, Alexander Brill, Steve P. Watson

**Affiliations:** ^1^ Institute of Cardiovascular Sciences College of Medical and Dental Sciences University of Birmingham Birmingham UK; ^2^ Centre of Membrane Proteins and Receptors (COMPARE) Universities of Birmingham and Nottingham The Midlands UK; ^3^ Department of Pathophysiology Sechenov First Moscow State Medical University (Sechenov University) Moscow Russia

**Keywords:** immune cells, inflammation, platelets, thrombo‐inflammation, thrombosis

## Abstract

Beyond their role in hemostasis and thrombosis, platelets are increasingly recognized as key regulators of the inflammatory response under sterile and infectious conditions. Both platelet receptors and secretion are critical for these functions and contribute to their interaction with the endothelium and innate immune system. Platelet‐leukocyte interactions are increased in thrombo‐inflammatory diseases and are sensitive biomarkers for platelet activation and targets for the development of new therapies. The crosstalk between platelets and innate immune cells promotes thrombosis, inflammation, and tissue damage. However, recent studies have shown that these interactions also regulate the resolution of inflammation, tissue repair, and wound healing. Many of the platelet and leukocyte receptors involved in these bidirectional interactions are not selective for a subset of immune cells. However, specific heterotypic interactions occur in different vascular beds and inflammatory conditions, raising the possibility of disease‐ and organ‐specific pathways of intervention. In this review, we highlight and discuss prominent and emerging interrelationships between platelets and innate immune cells and their dual role in the regulation of the inflammatory response in sterile and infectious thrombo‐inflammatory diseases. A better understanding of the functional relevance of these interactions in different vascular beds may provide opportunities for successful therapeutic interventions to regulate the development, progression, and chronicity of various pathological processes.

**Essentials**

- Platelet‐innate immune cell interactions regulate the development and outcome of thrombo‐inflammatory diseases
- Platelet activation potentiates innate immune cell recruitment, activation, and transmigration
- Platelets promote the progression and resolution of inflammation in a disease‐specific manner
- Platelet‐leukocyte interactions differentially regulate thrombo‐inflammation

## 1 INTRODUCTION

Platelets are small, anucleated cell fragments derived from megakaryocytes and known for their hemostatic functions at the site of vascular lesion. In the past 2 decades, multiple roles for platelets beyond hemostasis have been shown, including in vascular permeability, inflammation, infection, and tissue repair.^1^, ^2^, ^3^, ^4^, ^5^ More recently, a new role for platelets in the maintenance of vascular integrity at the site of inflammation was described, a process currently termed *inflammatory hemostasis*.^3^, ^6^, ^7^, ^8^, ^9^, ^10^ Both hemostatic and immunomodulatory functions of platelets are tightly regulated by environmental cues, in particular their interaction with the endothelium and innate immune system components.

The concept of thrombo‐inflammation was originally introduced to describe the role of platelets in the inflammatory response following cerebral ischemia‐reperfusion injury.^11^ It is currently more broadly used to describe various diseases regulated by the crosstalk between thrombosis and inflammation, such as deep vein thrombosis, stroke and atherosclerosis, and infectious diseases, such as sepsis. The common feature in thrombo‐inflammatory disease is interplay of the endothelium and immune and hemostatic systems. In these diseases, inflammation triggers thrombosis, which in return fuels the inflammatory response. Increased platelet‐leukocyte interactions on the inflamed endothelium, in thrombi, and in the blood are observed in thrombo‐inflammatory experimental models and in patients with atherosclerosis, acute coronary syndrome, ischemic stroke, deep vein thrombosis (DVT), and sepsis.^12^, ^13^, ^14^, ^15^, ^16^, ^17^ The level of circulating platelet‐leukocyte aggregates (PLAs) is suggested to be both a sensitive biomarker for platelet activation and a novel therapeutic target. For example, platelet activation and platelet‐monocyte aggregates in acute coronary syndrome are used as an early hallmark for acute myocardial infarction.^12^ Furthermore, platelet‐monocyte aggregates after myocardial infarction are more sensitive markers of platelet activation than P‐selectin.^13^ However, it is unclear whether these interactions are a cause, an active participant, or merely an epiphenomenon of the inflammatory response. In this review, we focus on established and emerging interactions between platelets and innate immune cells and discuss the evidence that links these interactions to function in disease states in different vascular beds, in particular in the maintenance of vascular integrity and inflammation.

## 2 PLATELETS SUPPORT CLASSICAL AND INFLAMMATORY HEMOSTASIS

At the site of vascular injury and under high shear conditions, platelets roll and adhere to the exposed extracellular matrix through glycoprotein Ib (GPIb)–von Willebrand factor (VWF) and glycoprotein VI (GPVI)–collagen interactions. Platelet activation leads to platelet integrin GPIIbIIIa activation, the release of granule contents including the feedback agonist ADP, and the formation of thromboxane A_2_ (TxA_2_), promoting recruitment of circulating platelets. While the formation of a platelet plug supports classical hemostasis, uncontrolled platelet activation leads to pathogenic thrombosis. Platelet granules are an important source of pro‐ and anti‐inflammatory mediators, for example, tumor necrosis factor‐α (TNF‐α); transforming growth factor‐β; chemokines, for example, RANTES, platelet factor 4 (PF4), and neutrophil activating peptide 2 (NAP‐2); endothelial cell modulators, for example, sphingosine‐1‐phosphate, and serotonin; and growth factors, for example, platelet‐derived growth factor (PDGF) and vascular endothelial growth factor (VEGF).^18^ The translocation of other proteins including P‐selectin and CD40‐ligand (CD40‐L) to the platelet surface favors their interactions with immune and endothelial cells promoting vascular permeability, thrombosis, and inflammation.^19^, ^20^, ^21^ Platelets also contain mRNAs and pre‐mRNAs that are translated upon platelet activation, with interleukin (IL)‐1β being the most studied.^22^ Platelet granule secretion occurs instantly following platelet activation, however, platelet IL‐1β is released a few hours after stimulation.

At the site of inflammation, platelets play a dual role in the maintenance of vascular integrity and inflammation.^10^ Platelet recruitment and activation increase vascular permeability, leukocyte recruitment, and edema through the release of chemokines and permeability factors with key roles for PF4, RANTES, VEGF, and serotonin.^10^, ^20^, ^23^ However, platelets also maintain integrity of the endothelial cell layer at sites of endothelial damage inflicted by neutrophil transmigration, thus preventing inflammatory bleeding.^24^ Inflammatory hemostasis is primarily GPIIbIIIa independent, although in certain inflammatory environments, such as ischemia‐reperfusion injury in the brain, GPIIbIIIa‐dependent aggregation can be required.^3^, ^10^ Many mechanisms underlie the protective role of platelets in inflammatory hemostasis including physical sealing of the damaged endothelium and secretion of soluble factors that tighten endothelial junctions.^9^ Interestingly, platelets adhere predominantly at the site of endothelial cell junctions during inflammation and guide neutrophils to their extravasation sites.^25^ Therefore, the location of platelet adhesion might be critical for prevention of leakage through endothelial junctions and limiting inflammatory bleeding.^6^, ^7^, ^9^ Unlike classical hemostasis, the roles of platelet receptors and secretion in inflammatory hemostasis depend on the vascular bed and the nature of the inflammatory stimulus.^7^, ^8^, ^9^ For example, platelet secretion is required to secure the endothelial barrier in the ischemic brain but not in the inflamed skin or lung.^26^ Similarly, platelet GPVI is required to maintain vascular integrity in the inflamed skin while GPIbα is required in the inflamed lung.^7^, ^8^, ^9^ Whether the difference is due to the nature and intensity of the inflammatory stimulus, endothelial cell heterogeneity, and/or the microenvironment is not known.

Following injury, restoration of vascular integrity, apoptosis, and recruitment of progenitor, stromal and immune cells are crucial for tissue restructuring, remodeling, and functionality. Platelets are involved in many of the stages of wound healing, and this is emphasized by the beneficial use of platelet‐rich plasma and platelet releasate in wound repair.^27^, ^28^ They promote progenitor cell recruitment; promote cytokine, chemokine, proangiogenic, and growth factor release; and support fibrin generation and modulate immune and stromal cell recruitment and activation.^29^ Platelets can also promote the migration of leukocytes and stromal cells through the secretion of metalloproteinase (MMP) and stimulation of MMP secretion from leukocytes and endothelial cells, promoting tissue remodeling and repair.^28^, ^30^ In addition, we have recently shown that impaired vascular integrity due to loss of platelet function can also be beneficial in skin tissue repair. Deletion of platelet C‐type lectin‐like receptor‐2 (CLEC‐2) and GPVI impairs vascular integrity during skin wound healing in mice and results in bleeding in the tissue.^31^ The leakage of blood cells and plasma in the tissue accelerates fibrin generation, inhibits inflammatory immune cell recruitment, and promotes angiogenesis. It is likely that the cause of bleeding and the related pathology define the beneficial potential of safeguarding vascular integrity.

## 3 PLATELETS PROMOTE LEUKOCYTE RECRUITMENT IN INFLAMMATION

**Figure 1:**
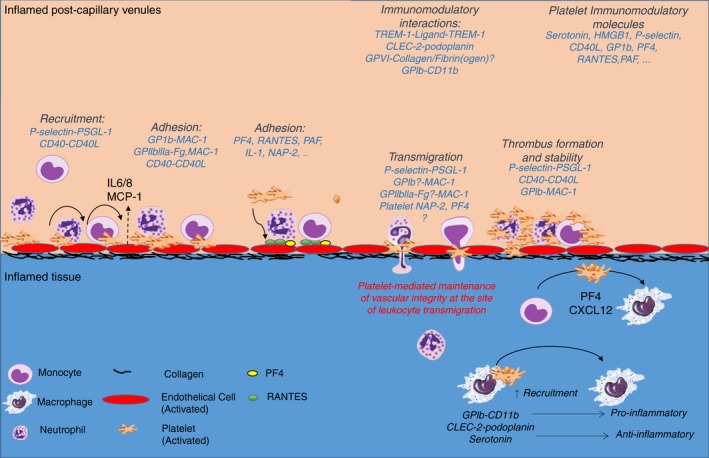
Platelets promote leukocyte recruitment, adhesion, and transmigration at the site of inflammation while maintaining vascular integrity. Under inflammatory challenge, platelets are the first cellular blood component adhering on the inflamed endothelium in small postcapillary venules. Adherent platelets recruit neutrophils and guide them in their adhesion and transmigration through cell‐cell interactions or release of chemokines on the endothelium. Activated platelets and neutrophils cooperate to recruit inflammatory monocytes with specific inflammatory stimuli such as MCP‐1 secretion required for efficient monocyte recruitment. At the site of leukocyte transmigration, platelets secure endothelial cell integrity to limit inflammatory bleeding. During inflammation, platelets promote the differentiation of monocytes into macrophages. Platelet receptors or releasate can shift macrophages towards a pro‐ or anti‐inflammatory phenotype. CLEC‐2, C‐type lectin‐like receptor‐2; GPVI, glycoprotein VI; IL‐1, interleukin‐1; GPIb, glycoprotein Ib; HMGB‐1, high‐mobility group box 1; MAC‐1, macrophage 1 antigen; NAP‐2, neutrophil activating peptide 2; PAF, platelet‐activating factor; PF4, platelet factor 4; PSGL‐1, P‐selectin glycoprotein ligand‐1; ; TREM‐1, triggering receptor‐expressed myeloid cells 1

Platelets and leukocytes use a multistep pathway to accrue at the site of inflammation, culminating in integrin activation and firm adhesion, with leukocytes undergoing further migration into the tissue. The recruitment of leukocytes to the inflamed endothelium is mainly observed in the postcapillary venules, probably due to the increased density of cell adhesion molecules and wall shear rate, with a key role for platelets in leukocyte recruitment in the brain venules.^32^ The interaction of platelets with leukocytes in the blood and at the site of activated and injured endothelium involves several receptor‐ligand pairs including P‐selectin–P‐selectin glycoprotein ligand‐1 (PSGL‐1), GPIbα–macrophage 1 antigen (MAC‐1, α_M_β_2_), GPIIbIIIa–MAC‐1 through fibrinogen, and CD40‐CD40L.^21^, ^32^, ^33^, ^34^ In the postcapillary venules, platelet P‐selectin is required for platelet and leukocyte recruitment, as shown in a large number of inflammatory models.^20^ The initial engagement of P‐selectin–PSGL‐1 induces rearrangement of the cytoskeleton of leukocytes and activation of β1‐ and β2‐integrins, leading to firm adhesion^35^, ^36^, ^37^ (Figure 1). Platelets can also interact indirectly with leukocytes through fibrinogen or VWF, and these interactions differentially regulate leukocyte recruitment and activation.^38^, ^39^ Under specific inflammatory challenges, such as ischemia and reperfusion injury, or injection of inflammatory molecules, such as angiotensin II, P‐selectin, and β2‐dependent leukocyte adhesion, is also observed in arterioles.^40^, ^41^ Although the major receptors and ligands have been intensively investigated, the implication of heterotypic cell‐cell interactions in different inflammatory contexts and organs are not well known.

Platelet‐mediated leukocyte recruitment is also potentiated through the activation of the complement system. Platelets express a large array of complement receptors such as cC1qR, gC1qR, C3aR, and C5aR and store complement proteins and regulators including C3 and factor H, respectively. Stimulated platelets activate the classical and alternative pathways of complement, leading to the deposition of opsonin C3b and the release of anaphylatoxins C3a and C5a, potent chemoattractants for innate immune cells.^42^, ^43^ In addition, platelet‐mediated complement activation promotes endothelial cell activation, upregulation of tissue factor, and release of inflammatory cytokines and chemokines, increasing leukocyte recruitment, activation, and thrombosis.

Several studies have shown the importance of platelets in neutrophil recruitment under inflammatory conditions including ischemia and reperfusion injury,^44^ DVT,^45^, ^46^ acute lung injury, 47 atherosclerosis,^48^ or abdominal sepsis.^49^ Following an inflammatory insult, neutrophils are rapidly mobilized from the bone marrow to the inflamed tissue in response to gradients of chemokines, complement components, inflammatory mediators, or microbial products.^50^ The initial interaction of P‐selectin on platelets with PSGL‐1 clusters on the neutrophil uropod plays a central role in neutrophil recruitment, activation of MAC‐1 and α_L_β_2_ (LFA‐1), and formation of neutrophil extracellular traps (NETs).^51^, ^52^ The activation of MAC‐1 also allows the direct binding of platelets through GPIb‐α and indirectly to GPIIbIIIa through fibrinogen, increasing neutrophil adhesion and transmigration.^33^, ^53^ These interactions physically tighten the bridge between neutrophils and endothelial cells and induce downstream signaling in neutrophils promoting their adhesion, crawling, and transmigration.^25^, ^54^ Platelet‐dependent neutrophil recruitment is further amplified by the secretion of inflammatory cytokines, chemokines, and growth factors, such as PF4, IL‐1, RANTES, β‐thromboglobulin, PDGF, platelet‐activating factor, CXCL7, migration inhibiting factor (MIF), TxA_2_ and serotonin, promoting endothelial cell activation, neutrophil recruitment, adhesion, and survival^55^, ^56^, ^57^, ^58^, ^59^ (Figure 1). Interestingly, single adherent platelets can promote the recruitment and nondirectional crawling of neutrophils. However, platelet secretion of NAP‐2 within thrombi generates a chemotaxis gradient to promote leukocyte directional migration through the thrombi in the veins.^19^ Some of these interactions occur in many vascular beds, but organ‐specific interactions also occur and contribute to the immunomodulatory functions of platelets and to inflammatory hemostasis.

During acute inflammation, monocyte release from the bone marrow is delayed compared to neutrophils, but they persist for longer and can infiltrate tissues and differentiate into macrophages.^60^ Neutrophils and monocytes share common phagocytic and inflammatory features; however, they differ in their chemotactic stimuli and receptors, release kinetics from the bone marrow, metabolic burst activity, and their interaction with the hemostatic system.^61^ Similar to neutrophils, monocyte recruitment to the vessel wall and their interaction with platelets involves P‐selectin, GPIb‐α, CD40L, and GPIIbIIIa.^21^ Platelets and neutrophils cooperate to promote subsequent stable inflammatory monocyte recruitment.^25^ Platelet‐mediated monocyte recruitment requires specific inflammatory stimuli such as monocyte chemoattractant protein 1 (MCP‐1), which induces efficient monocyte recruitment in the inflamed small postcapillary venules.^25^ Monocyte stable adhesion to the inflamed endothelium is further supported by platelet‐derived molecules, such as PF4, RANTES, and MIF, and also chemokines secreted from the vessel wall, such as MCP‐1.^62^, ^63^, ^64^ Therefore, the initial attachment of neutrophils and monocytes to the inflamed endothelium might not be selective, but some specificity can be acquired through interaction with chemokine receptors and proteins selectively expressed on leukocytes and on the endothelium of different vascular beds.^21^

## 4 PLATELET‐LEUKOCYTE INTERACTIONS DRIVE THROMBOSIS AND INFLAMMATION

Platelet‐immune cell interactions are critical for limiting pathogen growth and dissemination. However, uncontrolled activation and recruitment lead to thrombosis, tissue damage, and loss of organ function. Activated platelets also induce apoptosis of immune and stromal cells in many thrombo‐inflammatory diseases including stroke and sepsis. In mouse models of stroke and retinal inflammation, activated platelets induce apoptosis in primary murine neuronal cells, human neuroblastoma cells, and mouse embryonic fibroblasts via membrane‐bound Fas ligand.^65^ This form of nonimmunogenic cell death is required for organ remodeling and to limit chronic inflammation. However, during sepsis, the induction of the expression of apoptotic molecules such as granzyme B due to alteration in both platelet and megakaryocyte mRNA profiles increases lymphotoxicity in splenocytes and apoptosis in multiple organs and decreases survival rate.^66^ This shows that similar mechanisms differentially regulate thrombo‐inflammation and disease‐specific targeting is required for successful treatment.

### 4.1 Platelet‐neutrophil interaction

Neutrophils confer immune protection during infection through the secretion of antimicrobial proteins, such as defensins, elastase, and myeloperoxidase; the release of NETs; reactive oxygen species (ROS) generation; and pathogen phagocytosis.^67^ Neutrophil activation also induces inflammation‐mediated thrombosis through different pathways including the upregulation of tissue factor, NETosis, and the release of cathepsin G and elastase, and supports thrombus stability through the interaction of GPIb‐α and MAC‐1, or CD40L.^45^, ^68^, ^69^, ^70^, ^71^, ^72^, ^73^, ^74^ MAC‐1 interacts with adherent platelets through GPIb‐α mediating firm adhesion of leukocytes. Furthermore, this interaction supports thrombosis as evidenced by a delay in thrombus formation after carotid and mesenteric injury in MAC‐1 deficient mice or by blocking MAC‐1–GPIb interaction.^72^, ^73^ The MAC‐1–GPIbα and shear‐dependent MAC‐1 interaction with fibrinogen promotes neutrophil adhesion, transmigration, and thrombus formation, with platelet‐derived protein disulfide isomerase reducing the allosteric disulfide bonds in GPIbα and increasing platelet–neutrophil interaction.^34^, ^38^, ^73^, ^75^, ^76^, ^77^ CD40L (soluble or membrane bound) upregulated on activated platelets interacts with CD40 on immune cells and promotes thrombosis and inflammation.^72^, ^74^ Moreover, the interaction with adherent platelets increases the activation of neutrophils by IL‐8, c5a, and other agonists and supports formation of homotypic highly embolic aggregates.^78^, ^79^ P‐selectin and CD40L also contribute to thrombosis, as shown by alteration in thrombus formation and stability by inhibiting P‐selectin or CD40L, respectively.^74^, ^80^

**Figure 2:**
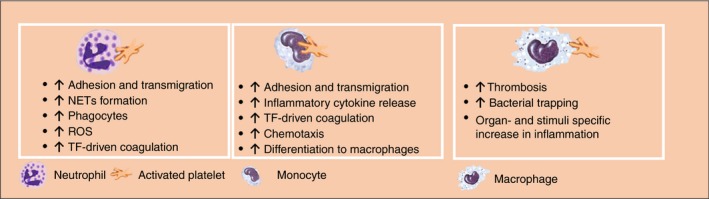
Platelet‐innate immune cell interactions modulate thrombo‐inflammation. Platelets interact with neutrophils, monocytes, and macrophages and modulate their effector functions. These interactions promote inflammation and thrombosis through different mechanisms which regulate the development and progression of thrombo‐inflammatory diseases. NETs, neutrophil extracellular traps; ROS, reactive oxygen species; TF, tissue factor

**Figure 3:**
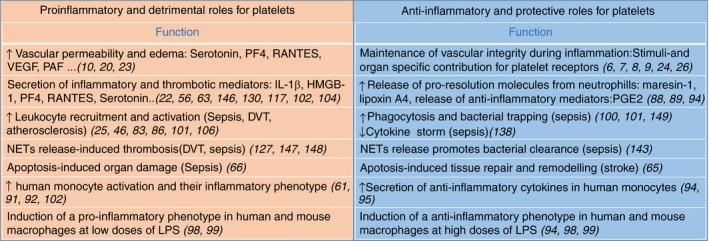
Dual role of platelets in thrombo‐inflammation. Platelets promote inflammation and organ damage by increasing vascular permeability, leukocyte recruitment, and activation. Platelets also contribute to the maintenance of vascular integrity, the anti‐inflammatory response and tissue repair. Many of these roles are disease and organ specific. DVT, deep vein thrombosis; HMGB‐1, high‐mobility group box 1; IL, interleukin; LPS, lipopolysaccharide; NETs, neutrophil extracellular traps; PAF, platelet‐activating factor; PF4, platelet factor 4; PGE2, prostaglandin E2; VEGF, vascular endothelial growth factor

Platelet interaction with neutrophils boosts their immune functions by increasing ROS generation, phagocytosis, and NET formation^34^, ^81^ (Figure 2). The interaction of activated neutrophils with platelets increases the release of sCD40L, leading to both increased ROS generation in platelets and neutrophils via 2 independent receptors, CD40 and GPIIbIIIa.^74^ In return, ROS and NETs support platelet‐neutrophil interaction, platelet activation, and vascular inflammation. Platelets also express receptors that are not required for platelet‐neutrophil interactions but regulate neutrophil activation. For example, the triggering receptor‐expressed myeloid cells 1 ligand on platelets increases neutrophil effector functions without altering platelet neutrophil complexes.^82^ More recently, the platelet immunoreceptor tyrosine‐based activation motif (ITAM) receptors CLEC‐2 and GPVI have been shown to regulate platelet interaction with neutrophils during infection and inflammation.^3^, ^83^, ^84^, ^85^, ^86^ GPVI promotes platelet‐neutrophil interaction during pneumonia and increases host defense.^87^ However, recent evidence suggests that platelet‐neutrophil interaction can also promote the resolution of inflammation through the secretion of prorepair molecules. The protective role of platelet‐neutrophil interaction was shown in murine models of acute respiratory distress syndrome and postischemia reperfusion injury in the mesenteric artery through the release of proresolution mediators such as maresin‐1 and lipoxin A4, respectively.^88^, ^89^ These studies show that platelets exert both protective and detrimental roles in thrombo‐inflammation, and it is likely that the balance between proinflammation and proresolution functions is highly dependent on the stage and the nature of the disease as well as the microenvironment (Figure 3).

### 4.2 Platelet‐monocyte/macrophage interactions

Platelets were also shown to modulate the effector functions of monocytes by inducing the activation of the nuclear factor kappa‐light‐chain enhancer of activated B cells (NF‐κb) and cyclooxygenase‐2 and the synthesis of inflammatory mediators, such as IL‐8 and MCP‐1^61^ (Figure 2). Thrombin‐activated platelets bind to monocytes through P‐selectin–PSGL‐1 axis. Blocking P‐selectin‐PSGL1 interaction however does not inhibit platelet‐mediated early Ca^2+^ mobilization in monocytes and their inflammatory phenotype suggesting distinct functions of P‐selectin‐PSGL1 in monocyte recruitment and activation.^90^ It is possible that early platelet‐mediated monocyte activation does not require monocyte attachment to platelets but is crucial for sustained activation. Indeed, enhanced chemokine synthesis requires P‐selectin–PSGL‐1 interaction for subsequent activation by RANTES.^91^ Platelet‐derived high‐mobility group box 1 (HMGB‐1) promotes human and mouse monocyte recruitment through receptor for advanced glycation end products (RAGE) and inhibits apoptosis through toll‐like receptor 4 (TLR‐4).^92^ These interactions also promote thrombosis through the release and accumulation of monocytes‐derived tissue factor‐bearing microparticles leading to a proinflammatory and prothrombotic phenotype in mice.^93^

Human collagen‐activated platelets were also shown to exert anti‐inflammatory functions on human monocytes by increasing the anti‐inflammatory cytokine IL‐10 and reducing TNF‐α in the absence of stimuli partly through the secretion of prostaglandin E2.^94^ Activated platelets also promote an anti‐inflammatory phenotype and IL‐10 production in human monocytes in presence of inflammatory stimuli (eg, high‐dose lipopolysaccharide [LPS], human thyroglobulin, intact *Porphyromonas gingivalis*).^95^ The anti‐inflammatory effect of activated platelets is mediated by platelet secretion, in particular CD40‐L as blocking CD40L using selective antibody counteracts the effect of platelets.^95^ Activated platelets differentially regulate the expression on monocyte cytokines and chemokines and their subsequent functions in inflammation.

During acute and chronic inflammation, platelets promote monocyte survival and differentiation into macrophages, partly through the secretion of chemokines such as CXCL‐12 and PF4.^96^, ^97^ In human monocyte‐derived macrophages, activated platelets promote LPS‐mediated inflammatory cytokine release (high‐dose LPS), which is largely dependent on platelet secretion.^98^ Using mice bone marrow–derived macrophages in vitro*,* activated platelets induces IL‐10 secretion from noninflamed macrophages and decreases TNF‐α release.^94^ At low doses of LPS, platelets promote TNF‐α secretion from macrophages but inhibit macrophage‐dependent inflammation at a high dose of LPS and during experimental bacterial peritonitis.^99^ How platelets sense the intensity of the inflammatory signal to regulate their immune functions is not known. Moreover, activated platelet enhances macrophage phagocytosis of *Staphylococcus aureus* (*S aureus*) independently of cell contact through the release of IL‐1β, increasing antimicrobial function of macrophages and protection against *S aureus* infection.^100^ In vitro, the anti‐inflammatory effect of platelets in the presence of high dose of LPS is independent of platelet secretion, suggesting a key role for membrane receptors. We have recently shown that platelet ITAM receptor CLEC‐2 is a key regulator of macrophage activation and recruitment in a mouse model of acute respiratory distress syndrome and polymicrobial peritonitis.^83^, ^86^ The protective role of CLEC‐2 is dependent on its interaction with podoplanin upregulated on inflammatory macrophages. Moreover, GPIb‐CD11b interaction was also shown to polarize monocytes toward a proinflammatory phenotype and to promote inducible nitric oxide synthase–positive macrophage recruitment to the infected peritoneum and increases bacterial clearance.^101^ How platelets exert both a pro‐ and anti‐inflammatory phenotype is not fully known. Differential platelet secretion might be related to these roles, although a single protein can promote pro‐ and anti‐inflammatory roles in different cells. For example, serotonin upregulates NF‐κB activation in monocytes^102^ but polarize macrophages toward an anti‐inflammatory phenotype.^103^ Recently, injection of immune complexes in transgenic FcγRIIA mice was shown to mediate platelet activation and the release of serotonin leading to neutrophil activation–dependent anaphylactic shock.^104^ These studies show the bidirectional beneficial or detrimental roles for platelet‐leukocyte interactions, making this another example of tissue‐, stimuli‐, and timing‐dependent regulatory functions for these interactions.

## 5 THE RELEVANCE OF PLATELET‐LEUKOCYTE INTERACTIONS IN THROMBO‐INFLAMMATORY DISEASES

The contribution of platelet‐leukocyte interactions to thrombo‐inflammation has been extensively studied in the last decade with recognition that the underlying mechanisms are tissue/organ specific. Below, we illustrate some known roles of these interactions in different vascular beds and in response to different insults, and discuss the involvement of both common and disease‐specific pathways in regulation of thrombo‐inflammation.

## 6 ATHEROSCLEROSIS

Atherosclerosis is a thrombo‐inflammatory disorder involving inflammatory and immune responses to oxidized lipids, endothelial dysfunction, and the formation of an atherosclerotic plaque. At the site of atherosclerosis, leukocytes and platelets accumulate and promote plaque growth and progression and eventually destabilize the endothelial layer leading to plaque rupture.^105^, ^106^, ^107^, ^108^ Platelet and leukocyte recruitment promote atherosclerosis as depletion of platelets, neutrophils or monocytes reduces plaque size.^106^, ^107^, ^108^ In severe atherosclerosis, platelet recruitment and adhesion preceded the development of atherosclerotic lesions followed by leukocyte recruitment to the arterial vasculature.^106^ On the intact plaque, platelets are recruited through GPVI‐laminin interaction promoting atheroprogression.^109^ At the site of fissured lesions, plaque rupture triggers platelet recruitment through GPVI‐collagen interaction. Inhibition of GPVI extracellular domain or downstream signaling inhibits thrombus formation on atherosclerotic plaque in vitro.^110^, ^111^

Platelet activation significantly contributes to the pathogenesis of atherosclerosis and chronic vascular inflammation, independently of atherothrombosis. They promote the uptake of oxidized low‐density lipoproteins (OxLDLs) by monocytes and macrophages,^112^ increase monocyte recruitment and adhesion to the inflamed or atherosclerotic endothelium,^48^ and secrete cytokines and chemokines, increasing plaque and possibly systemic inflammation. Activated platelets promote monocyte recruitment directly through the interaction of P‐selectin with PSGL‐1 and CD40L–MAC‐1 and indirectly through the deposition of PF4 and RANTES on endothelial cells and monocytes or delivered in microparticles.^48^, ^113^, ^114^, ^115^, ^116^, ^117^ Moreover, PF4 was shown to downregulate atheroprotective genes in human macrophages and to increase OxLDL uptake by macrophages, exacerbating atherosclerosis.^118^, ^119^ Platelet PF4 forms heteromers with RANTES, resulting in increased monocyte adhesion to endothelial cells and disruption of this interaction inhibits atherosclerotic plaque formation in hyperlipidemic mice and in a mouse model of stroke.^120^, ^121^ Moreover, platelet‐dependent monocyte recruitment and activation may increase plaque instability, partly by promoting matrix metallopeptidase 9 production by monocytes.^122^ Platelet activation and secretion might also increase endothelial permeability and facilitate the accumulation of lipids within the vessel wall.

PLAs, and in particular platelet‐monocyte aggregates, are increased in patients with atherosclerotic vascular disease.^17^ The elevated number of circulating PLAs increases the risk of development of cardiovascular and cerebrovascular diseases associated with increased endothelium activation, suggesting a proinflammatory role of PLAs in atherosclerosis.^17^ Due to the role of platelets in inflammation and thrombosis, classical antiplatelet drugs are used to treat thrombotic events in arterial cerebrovascular and cardiovascular thrombosis including atherosclerosis.^21^, ^123^ In particular, P2Y_12_ inhibitors have additional anti‐inflammatory properties associated with a decrease in platelet P‐selectin, PLAs, and soluble CD40L and RANTES.^17^ Despite the antithrombotic and anti‐inflammatory effects, these drugs do not prevent the progression of established atherosclerosis in patients. More recently, other therapies that disrupt PLAs were developed, including inhibitors of P‐selectin, PSGL‐1, CD40L, and GPIb and their beneficial or harmful outcome in atherosclerosis was recently reviewed.^21^ While some of these inhibitors showed promising results in experimental models, results from some clinical trials have been disappointing while others are still ongoing. Moreover, the role of membrane receptors and soluble mediators might require combined therapies to improve clinical outcomes. The heterogeneity of the disease and the engagement of multiple receptors throughout disease progression might account for the differences observed between patients and experimental models.^21^ Therefore, the use of biomarkers for different disease stages might increase the possibility of selective drug targeting, with reduced side effects.

## 7 DEEP VEIN THROMBOSIS

DVT is a multifactorial disease, in which the reduction of blood flow, hypoxia, and endothelial and stromal cell activation contribute to thrombus development in veins. DVT is most commonly developed in the legs under the muscular fascia of the limbs or in the central deep veins. DVT causes and pathogenesis may differ depending on genetic and environmental factors. It is important to note that the location and stability of the thrombi rather than thrombus size is associated with worse outcome due to the life‐threatening increased risk in pulmonary embolism.^124^, ^125^ Moreover, the damage inflicted to the vessel wall promotes a postthrombotic syndrome and increases the risk of recurrent DVT. During experimental DVT in mice triggered by the ligation of the inferior vena cava, platelets, and leukocytes are recruited to the vascular wall preceding thrombus formation.^46^, ^126^ Platelets are recruited as single cells or small aggregates, directly binding to the vessel wall and forming heterotypic aggregates with leukocytes via GPIbα. Whereas monocytes support DVT predominantly by providing tissue factor, which triggers blood coagulation, neutrophils promote thrombosis by releasing NETs.^127^ The factor triggering NETosis in the sterile environment inside the blood vessel has been proposed to be platelet‐derived HMGB‐1. The effect of HMGB‐1 is potentiated via the P‐selectin–PSGL‐1 axis,^128^ with P‐selectin–deficient mice being protected against DVT.^46^, ^129^, ^130^ In addition to promoting NETosis, platelet‐originated HMGB‐1 also increases neutrophil and monocyte sequestration at the venous wall, increasing local inflammation. More recently, complement activation was shown to regulate the development of DVT in mice, with complement components displaying distinct roles in thrombus formation. C3 activation leads to platelet and fibrin deposition, whereas C5 increases tissue factor expression on monocytes and precipitates fibrin generation, independently of platelets, promoting thrombo‐inflammation.^131^

An increase in PLAs is observed in patients with venous thrombosis.^16^ In particular, the increase in circulating platelet‐neutrophil aggregates has been shown to correlate with platelet activation in individuals with DVT,^132^ with the ROC revealing that the level of platelet‐neutrophil aggregates represents a risk factor for venous thrombosis. Platelet‐monocyte aggregates are also increased after surgery in patients with venous thromboembolism (VTE), and their count correlates with plasma levels of an important systemic proinflammatory marker, C‐reactive protein.^133^

Anticoagulants are the common mainstay of treatment of DVT, although thrombolysis, mechanical thrombectomy, and angioplasty are also used. Antiplatelet drugs, in particular aspirin, were shown to reduce the risk of primary thromboembolism and the recurrence of secondary VTE when used as a long‐term secondary preventive strategy in patients with VTE following an initial anticoagulant treatment.^134^, ^135^, ^136^

Thus, although the activation of the coagulation system drives venous thrombosis, the interaction of platelets with innate immune cells, both in the flowing blood and at the vessel wall, directly contribute to the initiation and progression of DVT and the associated inflammatory response.

## 8 SEPSIS

Platelets express a wide range of complement receptors, pattern recognition receptors, in particular toll‐like receptors and Fc receptors, providing the ability to sense and respond to endogenous and exogenous inflammatory and infectious signals and initiate an immune response.^137^ Following activation, platelets secrete a large array of antimicrobial and immunomodulatory molecules that can directly kill pathogens and/or enhance immune cell differentiation and activation. Platelet depletion and thrombocytopenia are associated with worse outcome, suggesting their protective role in sepsis.^85^, ^138^ In mouse models of bacterial peritonitis, early platelet transfusion is protective through the regulation of macrophage activation.^99^, ^101^ Platelet transfusion attenuates thrombocytopenia, decreases plasma levels of inflammatory cytokines such as TNF‐α and IL‐6, and improves survival.^99^ Platelet transfusion was also shown to increase inflammatory macrophage recruitment to the infected peritoneum and improve bacterial clearance through GPIb‐CD11b interaction.^101^ It is possible that platelet transfusion dampens the systemic inflammatory response partly through sequestration of cytokines released from activated immune cells and the regulation of immune cell activation.^83^, ^85^, ^86^, ^101^

During sepsis, platelets contribute to leukocyte recruitment and activation through both direct interaction and via secretion supporting immune cell recruitment and pathogen clearance, with an organ and pathogen role for platelet receptors and secretion.^139^, ^140^ Deficiency in these interactions reduces PLAs in bacterial sepsis and increases bacterial growth in an organ‐ and insult‐dependent manner.^85^, ^87^, ^139^, ^141^, ^142^ Interestingly, the protective role of platelet receptors depends on the pathogen and the site of infection. Following gram‐negative bacterial infection in mice, LPS binding to TLR4 on platelets induces NET release from neutrophils and sequesters bacteria within the vasculature, in particular in pulmonary capillaries and liver sinusoid.^143^ NET release is also induced by P‐selectin,^128^, platelet release of HMGB‐1,^144^ β1‐defensins,^145^ and other interactions. Moreover, LPS binding to TLR4 on platelets induce the shedding of IL‐1β rich microparticles increasing endothelial cell activation and propagating the inflammatory response.^146^ Platelets and NETs also induce disseminated intravascular coagulation and alter organ functions.^147^ Targeting NETs in sepsis must be finely tuned to limit organ damage but contain bacterial growth and spreading. This is evidenced by the beneficial role of the delayed injection of DNase in cecal ligation and puncture models compared to the detrimental effect observed at an earlier time in sepsis.^148^

The liver has specialized macrophages, known as Kupffer cells, that line the walls of the sinusoids and are thus constantly exposed to the blood flow. Under physiological conditions, platelets transiently interact with intravascular Kupffer cells via GPIbα‐VWF as part of the innate immune surveillance system of the liver.^149^ The interaction of platelets with Kupffer cells is stabilized in the presence of bacteria such as *Bacillus cereus* and methicillin‐resistant *Staphylococcus aureus* through GPIIbIIIa‐mediated platelet adhesion. Platelet stable adhesion increases neutrophil recruitment to the liver sinusoids and participates in the host response against the pathogen.^149^ More recently, the platelet ITAM receptors CLEC‐2 and GPVI have been shown to regulate platelet interaction with neutrophils and macrophages during infection.^3^, ^83^, ^84^, ^85^, ^86^ GPVI promotes platelet‐neutrophil interaction during pneumonia increases host defense against pathogens.^87^ The interaction of podoplanin upregulated on inflammatory macrophages has been shown to promote CLEC‐2–mediated platelet aggregation in vitro^150^ and in vivo.^84^ In a mouse model of systemic *Salmonella typhimurium* infection, platelet CLEC‐2 interacts at the site of vascular breaches with podoplanin‐positive macrophages and drive pathogenic thrombosis in the liver.^84^ These thrombi did not contain *Salmonella* and peaked when bacteria in the blood and tissues were declining. Deletion of platelet CLEC‐2 abolished liver thrombosis without altering bacterial count.^142^ In contrast, the CLEC‐2‐podoplanin axis inhibits inflammation in mouse models of LPS‐induced acute respiratory distress syndrome and cecal ligation and puncture without altering thrombosis.^83^, ^85^, ^86^ The inhibition of CLEC‐2–podoplanin interaction exacerbates the cytokine storm and impairs macrophages recruitment to the infected peritoneum increasing bacterial load. Therefore, the nature and receptors involved in PLAs formation in sepsis depends on the organ involved, the time course of the infection, and the insult.

PLAs are also increased in septic patients and exhibit a reciprocal relationship to survival in patients developing multiple organ failure, probably due to an increase in sequestration.^151^ Indeed, platelet‐neutrophil aggregates are elevated during the early phases of sepsis but significantly decrease with sepsis progression.^151^ An increase in platelet‐monocyte aggregates is associated with increased mortality in older septic patients but not in younger patients.^152^ Therefore, targeting platelet‐immune cell interaction in sepsis plays either beneficial or detrimental roles according to the model, disease stage, and associated comorbidities. Similarly, the role of PLAs depends on the stage of the infection, the immune cells involved, and their contribution to pathogen clearance, and organ damage.

## 9 CONCLUSION

There is compelling evidences for a functional relevance of platelet‐leukocyte interactions in thrombo‐inflammatory diseases. Many antiplatelet drugs or inhibitors targeting selective interactions between platelets and leukocytes were shown to modulate clinical outcome in experimental models. The protective or deleterious effect of these interactions on the clinical outcome largely depends on the pathophysiological context and disease stage.^21^, ^153^ This is not surprising based on recent studies showing different thrombo‐inflammatory mechanisms in different organs and at different disease stages. Moreover, the dynamic of platelet and leukocyte engagement in the arterial and venous inflamed vasculatures might require selective inhibitors for successful therapies in different diseases. GPVI inhibitors have emerged as promising targets to reduce thrombus formation on atherosclerotic plaques and possibly in other atherothrombotic diseases.^111^ CLEC‐2 is a potential target in the inflamed venous system including DVT and *Salmonella*‐mediated thrombosis in the liver. However, the clinical efficacy in thrombo‐inflammatory disease in human is still to be proven. A better understanding of the dynamics of thrombo‐inflammatory mechanisms in different diseases is critical for successful therapeutic intervention with selective inhibitors required at different stage of the disease and in different organs.

## RELATIONSHIP DISCLOSURE

The authors have no conflicts of interest to disclose.

## AUTHOR CONTRIBUTION

JR, JHB AB, and SPW wrote the manuscript.
